# Supplementary material for: Quality of life of COVID 19 patients after discharge: Systematic review
Source: PLoS One. 2022 Feb 16;17(2):e0263941. doi: 10.1371/journal.pone.0263941 (PMC8849513; doi:10.1371/journal.pone.0263941)
Supplement: Attachment — Submitted filename: Responses to the reviewer comments.docx [file pone.0263941.s003.docx]

**Responses to the reviewer comments**

| **Reviewer comment** | **Response** |
| --- | --- |
| Some fine tuning of the contents is needed to strictly match the requirements of PLOS manuscript guidelines. | Rearranged the contents according to the journal requirement. |
| The one reservation I have, is the issue of ethical consideration. Though rarely discussed in the context of systematic reviews, I believe the authors should address the matter as it is associated with potential conflicts of interest and issues of data representation. | Statement on ethical consideration was added to the research article (See lines: # 125 to 127). |
| Additionally, it would be better to expand the conclusion segment to highlight how your review impacts current public health practice. | Conclusion segment was expanded highlighting the impact of the findings to current public health practice (See lines: #299 to 306). |
| Grammatical errors/ incomplete sentences; ¶& both the pilcrow and ampersand symbols represent equal contribution to the work, you could stick to one. | Corrected (See the title page). |
| Will the use of female sex be more suitable compared with female gender? | Female sex was used instead of female gender throughout the manuscript. |
| In the background, clinically stable COVID 19 patients can also be presented with depressive symptoms and lower quality of life following recovery (doesn't read well). There are at least 150 instruments exist to assess a person's QOL...(check sentence). In this context, the research evidence on the QOL of COVID19 patients following discharge or recovery has grown at very rapidly. In the Quality assessment of the studies section; Out of 14 criteria used to assess the quality of a study were met by all the studies. (Check wording). | Corrected (See lines: #47 to 49; 52; 55 to 56 and 171). |
| Is there a numerical correlation between the EQ-5D subscales and the SF-36 subscales that you could include in the discussion? | A study done by Rowen et al, suggested that models mapping the SF-36 onto the EQ-5D have similar predictions across inpatient and outpatient setting and medical conditions.  This fact also included into the discussion (See lines: # 264 to 266). |
